# Proper bile duct flow, rather than radical excision, is the most critical factor determining treatment outcomes of bile duct cysts

**DOI:** 10.1186/s12876-018-0862-3

**Published:** 2018-08-23

**Authors:** Hong-Tian Xia, Tao Yang, Yang Liu, Bin Liang, Jing Wang, Jia-Hong Dong

**Affiliations:** Hospital and Institute of Hepatobiliary Surgery, Chinese PLA General Hospital, Chinese PLA Medical School, Beijing, 100853 China

**Keywords:** Bile duct cyst, Biliary-enteric flow, Biliary reconstruction, Biliary flow reestablishment, Total excision, Long-term biliary function

## Abstract

**Background:**

The purpose of this study was to compare the impact of the extent of excision and the patent bile duct flow on treatment outcomes of bile duct cysts (BDCs).

**Methods:**

We retrospectively analyzed the records of 382 patients who received surgery for BDCs from January 2005 to December 2014.

**Results:**

For Type Ia cysts, proper bile flow was associated with good long-term treatment outcomes with a greater level of significance (*p* < 0.001) than complete excision (*p* = 0.012). For Type IVa cysts, proper bile flow, but not complete excision, was associated with good long-term outcomes (*p* < 0.00001). In addition, 96.3% (104/108) of Type IVa patients with proper bile flow had no late complications and good biliary function, while no patient without patent bile flow had a good clinical outcome. For Type Ic cysts, 92 patients who received partial excisions had good outcomes when proper bile flow was restored. Regression analysis revealed that the absence of proper bile flow, in comparison to incomplete excision, is a greater risk factor for poor long-term treatment effects for Type Ia and Type IVa cysts.

**Conclusions:**

Compared to complete excision, the establishment of proper bile flow exerted a greater impact on improving long-term clinical outcomes after BDC surgery.

**Electronic supplementary material:**

The online version of this article (10.1186/s12876-018-0862-3) contains supplementary material, which is available to authorized users.

## Background

Bile duct cyst, or choledochal cyst (CC), has been considered to be a rare disease that mostly occurs in children in western countries. However, the number of adult patients with CCs has been increasing in recent years due to improvements of noninvasive hepatobiliary imaging, with the reported incidence reaching up to 70% [1–6]. Adult CCs are particularly frequent in Asian countries, with a frequency of 1 per 5000 in China and 1 per 1000 in Japan [7–9]. Long-term postoperative complications of adult CCs can be as high as 80% [3–6, 10, 11]. Cholangiocarcinoma is also more frequent in adult CC patients [4, 6]. The frequency of cholangiocarcinoma increases with age, and is approximately 0.42% in children under 18 years of age, 11.4% in adults, and 50% by the age of 50 [12–14].

Complete/radical cyst excision plus Roux-en-Y hepatojejunostomy has substantially reduced the incidence of postoperative long-term complications and cancer in patients with CCs [10, 15–18]. As such, radical excision of CCs is considered the gold standard for the treatment of Todani Type I and Type IV CCs, and it is believed that incomplete cyst excision is associated with poorer clinical outcomes and a greater risk of malignancy [7, 19]. We have also addressed the issue of surgical approach at our institution, and our findings support the effectiveness of complete excision in reducing the rate of complications and cancer [8, 15]. However, frequently there is no histological boundary between the lesion and normal bile duct, and the so-called radical excision is based solely on the morphology assessed by visual inspection during surgery. Clinically, complete excision is not always achievable, especially in patients with widespread intrahepatic cysts, as is often found with Todani Type Ic and some Type IVa cysts [20]. As such, the rate of long-term postoperative complications remains high with an overall rate of 29.7% in adults, and 68.7% in patients of Type IVa cysts [3, 6, 11, 21].

At our institution we have treated a large number of patients who were previously treated at other institutions with or without radical excisions who required re-operation. We have observed that radical excision may not be the most critical factor determining long-term postoperative outcomes. We also observed that when radical excision could not be achieved in patients with Type I and IVa cysts, good long-term postoperative outcomes could still be obtained if patent bile duct flow was established.

The purpose of this study was to retrospectively analyze 382 cases of Todani Type Ia, Ic or IVa bile duct cysts to determine the importance of patent bile duct flow in comparison to the extent of excision (complete or partial) with respect to long-term postoperative outcomes.

## Methods

### Patients

The study was approved by the Institutional Review Board of the Chinese PLA General Hospital. All patients provided written informed consent for all procedures performed.

We respectively reviewed the records of all adult patients with a definite diagnosis of a Todani Type Ia, Type Ic, or Type IVa bile duct cyst who had surgical resection at our hospital during the period from January 2005 to December 2014. All patients had laboratory testing including white blood cell count (WBC), alanine aminotransferase (ALT), aspartate aminotransferase (AST), alkaline phosphatase (ALP), and gamma-glutamyl transferase (GGT). The diagnosis of a Todani Type Ia, Ic, or IVa bile duct cyst was made by imaging studies that included abdominal ultrasonography, computed tomography (CT), and magnetic resonance cholangiopancreatography (MRCP). Postoperatively the diagnosis was confirmed by histopathological examination of the surgical specimen. Patients with biliary dilatation due to bile duct obstruction or bile duct stones were excluded from the study. Patients who had a reoperation, or with a follow-up duration less than 12 months were also excluded.

### Surgical methods

The surgical treatment of patients with a Todani Type Ia bile duct cyst included complete excision or partial resection (due to hilar bile duct stricture) followed by a Roux-en-Y hepaticojejunostomy. In patients with a hilar bile duct stricture, a hilar ductoplasty was performed prior to the Roux-en-Y hepaticojejunostomy. For all Type Ic cysts, the surgery for all patients was the removal of the bile duct cysts plus Roux-en-Y hepaticojejunostomy. For Type Ia cysts, all patients were undergone the removal of the bile duct cysts, plus Roux-en-Y hepaticojejunostomy and hepatic hilar angioplasty.. Complete or partial excision was determined morphologically, rather than pathologically.

For Type IVa cysts, there were two types of surgeries. Surgery I included the removal of the extrahepatic bile duct cyst, the hepatic hilar angioplasty and Roux-en-Y hepaticojejunostomy. Surgery II included all operations of Surgery I plus partial hepatectomy.

Establishment of proper bile flow was defined according to the following criteria: 1) there was no relative bile duct constriction proximal to the anastomosis of the cholangioenterostomy; 2) the diameter of the bile duct anastomosis was greater than the diameter of the proximal intrahepatic bile duct, and there was no sign of intrahepatic cholestasis after biliary enteric anastomosis; 3) the absence of an anastomotic stricture and of intrahepatic biliary dilatation confirmed by postoperative imaging; 4) postoperative serum ALT, AST, GGT, and ALP levels returned to normal.

During postoperative follow-up, local patients were evaluated at our institution. For patients from outside the city, follow-up took place at their local hospitals. If there were any abnormalities found in non-local patients, the patients were reassessed at our institution. During the first postoperative year, all patients were followed-up every 3 months and thereafter every 6 months. Beginning at 5 years after surgery, reexamination was performed annually. Reexamination included abdominal ultrasonography, CT, and MRI, as well as biochemical blood testing. The assessments performed at the local and nonlocal hospitals were the same.

Long-term surgical efficacy was based on the occurrence of long-term postoperative complications (late complications) and long-term postoperative biliary function, and was evaluated at each revisit during the full follow-up period (67.2 ± 29.7 months). Efficacy was evaluated based on the method by Moraca et al. [22], and the criteria developed by Lillemoe et al. [23]. Four categories were included: 1) excellent, normal biochemical examination without clinical symptoms or anatomical abnormalities; 2) good, no clinical manifestations of cholangitis with only a few small bile ducts with abnormal structures, normal or close-to-normal biochemical testing results, and no need for further intervention; 3) fair, presence of mild anatomical abnormalities of the bile ducts, clinical manifestations of cholangitis with onset < 3 times per year, and symptoms that were amenable to conservative treatment such as antibiotics; 4) poor, recurrent cholangitis and abnormal anatomical structures of the bile ducts, bile duct strictures, and stones that did not respond to conservative treatment and required a reoperation [21]. The categorizations were consistent with those used in previous studies [9].

Late complications (e.g., recurrent cholangitis, bile duct stones, biliary stricture, cancer) were defined as those occurring after hospital discharge, and complications were assessed throughout the course of the follow-up period.

### Statistical analysis

Continuous variables were expressed as mean ± standard deviation (SD), while categorical data were expressed as number (%). Data were analyzed with a chi-square test or Fisher’s exact test. All statistical analyses were performed using IBM SPSS version 20, with an alpha level of 0.05. Multiple logistic regression was performed to evaluate whether the extent of resection and the establishment of patent bile duct drainage were an independent risk factor for complications.

## Results

The demographic data, and preoperative clinical information of the 382 patients are presented in Table 1. The average age of the patients was 40.4 ± 12.0 years, and there were 298 females and 84 males.

**Table 1:** Demographic data and preoperative status

Characteristics	Data
Age (years)	40.4 ± 12.0
Gender	
Male	84 (22)
Female	298 (78.0)
Follow-up duration (months)	67.2 ± 29.7
Todani type	
Ia	166 (57.2%)
Ic	92 (24.1%)
Type IVa diffused	76 (19.9%)
Type IVa localized	48 (12.6%)

Data are expressed as mean ± standard deviation or number (percentage)

There were 37 patients who experienced different types of complications during hospitalization. The total complication rate was 9.69% (37/382), which was 7.75% (20/258) and 13.71% (17/124) for type I and IVa cysts, respectively. The main recent complications and the grading are shown in the Additional file 1: Tables S2 and S3. The treatment for bile and pancreatic leakage was to ensure patent flow. Abdominal infections were treated with antibiotics, while two cases were treated with ultrasound-guided puncture and drainage. Abdominal hemorrhage was mainly treated by hepatic arterial embolization, but two cases with poor embolization was treated with open hemostasis.

Four patients had cancer during the follow-up period of 46 to 88 months, the rate of postoperative cancer was 1%. Two cases were of type Ia and two were of type IVa cysts. Three had radical resection, but none had proper bile flow (for detail, see Additional file 1: Table S4).

The association of complete/partial excisions or proper bile flow with late complications and long-term biliary function in patients with Todani Type Ia bile duct cysts is shown in Table 2. In total, proper bile flow was achieved in 150 patients (90.4%). Consistent with previous reports, the extent of excision (complete vs. partial excision) was significantly associated with long-term postoperative clinical outcomes (aspects of late complications and biliary function) after Type Ia bile duct cyst surgery (*p* = 0.012). Patients who had a complete excision and a proper bile flow had a significantly lower rate of late complications (0% vs. 16.7%, *p* < 0.001), and a significantly higher rate of excellent biliary function outcomes (94.2% vs. 25%, *p* < 0.001) compared to patients with partial excision and proper bile flow. These data indicate that complete excision is important for achieving good outcomes in Type Ia bile duct cyst surgery.

**Table 2:** Late complications and long-term biliary function of patients with Type Ia bile duct cysts

	Complete excision+ proper bile flow (*n* = 138)	Complete excision only (*n* = 14)	Partial excision+ proper bile flow (*n* = 12)	Partial excision only (*n* = 2)	*p*-value (function of extent of excision)	*P*-value (function of proper bile flow)
Late complications	0	12 (85.7)	2 (16.7)	2 (100)	0.012	<.00001
Long-term biliary function					0.012	<.00001
Excellent	130 (94.2)	1 (7.1)	3 (25)	0		
Good	8 (5.8)	1 (7.1)	7 (58.3)	0		
Fair	0	10 (71.4)	2 (16.7)	0		
Poor	0	2 (14.4)	0	2 (100)		

Data are expressed as number of cases (percentage)

*P*-values were calculated based on a chi-square test to compare distributions of late complications and long term biliary function on complete excision and bile flow status

However, compared to complete excision, proper bile flow was associated with good long-term postoperative clinical outcomes with a greater level of significance (*p* < 0.001). While in the absence of patent bile flow, complete excision alone resulted in a high rate of late complications (85.7%), and poor long-term postoperative biliary function (excellent + good, 14.3%). Thus, compared to the extent of excision, the presence of patent bile flow appears to have a greater impact on the effectiveness of surgical treatment for Type Ia bile duct cysts (Table 2).

For Type IVa cysts, complete excision showed no significant association with good long-term postoperative outcomes (*p* = 0.285; Table 3). Rather, a significant association was found between proper bile flow and good long-term outcomes (*p* < 0.00001; Table 3). Of the 108 patients with proper bile flow, 104 (96.3%) had no late complications and had excellent or good biliary functions, while all 16 patients without proper bile flow had late complications and fair or poor biliary function. Thus, as long as proper bile flow is present, the extent of excision did not significantly influence the long-term postoperative outcomes for Type IVa cysts.

**Table 3:** Late complications and long-term biliary function of patients with Type IVa bile duct cysts

	Complete excision+ proper bile flow (*n* = 40)	Complete excision only (*n* = 4)	Partial excision+ proper bile flow (*n* = 68)	Partial excision only (*n* = 12)	*P*-value (function of excision level)	*P*-value (function of bile flow)
Late complications	1 (2.5)	4 (100)	3 (4.4)	12 (100)	0.285	0.00001
Long-term biliary function						
Excellent	36 (90)	0	57 (83.9)	0	0.285	0.00001
Good	3 (7.5)	0	8 (11.8)	0		
Fair	1 (2.5)	3 (75)	2 (2.9)	9 (75)		
Poor	0	1 (25)	1 (1.4)	3 (25)		

Data are expressed as number of cases (percentage)

*P*-values were calculated based on a chi-square test to compare distributions of late complications and long term biliary function on complete excision and bile flow status

Late complications and long-term biliary function of the 92 patients with Type Ic bile duct cysts who had a partial excision and proper postoperative bile flow are shown in Table 4. Late complications occurred in only 4.3% of the patients, and long-term biliary function was satisfactory in 95.7%.

**Table 4:** Late complications and long-term biliary function of patients with Type Ic bile duct cysts

	Partial excision+ proper bile flow (*n* = 92)	*P*-value
Late complications	4 (4.3)	N/A
Long-term biliary function		N/A
Excellent	80 (87.0)	
Good	8 (8.7)	
Fair	4 (4.3)	
Poor	0 (0)	

Univariate regression analysis was conducted to evaluate whether incomplete excision and the lack of proper bile flow were independent risk factors for late complications. As shown in Table 5, incomplete excision was a significant risk factor for late complications in Type Ia of bile duct cysts patients (*p* < 0.01 and 0.05, respectively), but not in Type IVa patients (*p* = 0.21). In comparison, lack of proper bile flow tended to be a greater risk factor for late complications in both Type Ia (*p* < 0.0001) and Type IVa bile duct cyst patients (*p* < 0.0001).

**Table 5:** Risk factor analysis of late complications on the two factors: incomplete excision and lack of proper bile flow (univariate regression analysis)

		incomplete excision				Lack of proper bile flow			
		Risk ratio	95% confidence limits		*P*-value	Risk ratio	95% confidence limits		*P*-value
Type	Ia	3.62	1.34	9.74	0.032	65.63	16.36	263.23	< 0.0001
	Ic								
	Iva	1.65	0.64	4.24	0.21	27	10.32	70.63	< 0.0001
Total		2.33	1.27	4.27	0.006	40.3	18.19	89.35	< 0.0001

The risk factor analysis for different type of surgery in patients with Type IVa cyst revealed no significant difference (Table 6).

**Table 6:** Risk factor analysis of late complications on the two factors for types of surgeries in Type IVa cysts

	Risk ratio	95% confidence limits		*P*-value
Late complications	1.2109	0.5416	2.7073	0.804
Long-term biliary function	1.184	0.5022	2.7915	0.797

Note: the clinical outcomes between two surgery methods (surgery I and II) for Type IVa cysts were compared

## Discussion

Long-term postoperative outcomes have remained unsatisfactory in adults patients with bile duct cysts [3–6, 21]. In China, the absence of patent bile duct drainage after bile duct cyst surgery is very common in both lower level hospitals and top level medical centers due to insufficient attention to this problem. Every year our medical center treats a great number of adult patients with bile duct cysts that require reoperation, and we have carried out more than 20 years of clinical research to improve the long-term treatment outcomes. We have published a series of reports on the topic [9, 24–26], including some that addressed the selection of surgical approaches and confirmed the importance of radical excision [8, 15]. During the 20 years of research, we have also noticed the crucial role of patent bile flow with respect to good long-term treatment outcomes, and therefore conducted the current study to specifically compare the role of these two factors.

Our results show that for Type Ia bile duct cysts, although complete excision of the lesioned bile duct significantly reduced the incidence of late complications and improved biliary function, the presence of proper bile flow impacted these clinical outcomes with a greater level of significance. More remarkably, for Type IVa cysts, as long as proper bile flow was ensured, complete or incomplete excision made no significant difference in terms of late postoperative complications and long-term biliary function. The regression analysis showed that the absence of proper bile flow is of greater risk compared to incomplete excision for late complications with Type Ia and IVa cysts; while incomplete excision is a risk factor for late complications in only Type Ia patients. In addition, patients with partial excision of Type Ic cysts had good clinical outcomes when proper bile flow was established.

These findings indicate that proper bile flow, rather than radical excision, is the most critical factor in reducing long-term postoperative complications and improving biliary function, although radical excision still plays an important role in the treatment of Type Ia cysts. These findings are of great clinical significance in that, if practiced widely and particularly in Asian countries, the improved surgical approach would greatly reduce the rate of long-term postoperative complications, lower the incidence of reoperation, and improve the surgical outcomes of bile duct cysts.

For complicated cases such as Type Ic and Type IVa cysts, radical resection of bile duct cysts cannot be easily achieved because of the wide distribution of cysts in the bile duct (Type IVa), or because there may be a fusiform dilatation and a pancreatobiliary malunion that may continue to the intrahepatic duct (Type Ic) [27]. We have shown that there were good long-term outcomes with these cysts when there was proper bile flow. Thus, assuring proper bile flow is essential in the surgical treatment of these types of bile duct cysts.

Of special note, in the absence of proper bile flow, patients who had complete cyst excision still had high incidences of late complications (85.7% and 100% for Type Ia and Type IVa cysts, respectively), and low rates of excellent and good long-term postoperative biliary function (14.3% and 0% for Type Ia and Type IVa cysts, respectively). These data further emphasize the importance of patent bile duct drainage, yet complete removal of the cysts alone does not necessarily guarantee proper bile flow into the gastrointestinal tract. The anatomical variants and the characteristics of bile duct cysts may lead to difficulties during hepaticojejunostomy, and occasionally cause postoperative biliary stricture, thereby resulting in bile stasis and cholangitis [15].

The issue of patent bile flow has been addressed previously. Todani et al. reported that free bile drainage is necessary to prevent ascending cholangitis [28]. Lenriot et al. showed that a long-lasting cure for bile duct cysts in adults can be accomplished if the following three conditions are met during the operation: eradication of pancreatic reflux, a decrease of the malignancy risk by reducing the common sites of malignant transformations, and restoration of normal bile flow [21]. Our practice is basically consistent with these studies. However, we specifically stress that to ensure proper bile flow, there should be no cholestasis or relative strictures at the anastomotic stoma. This is achieved by cholangioplasty at the porta hepatis and partial hepatectomy. Moreover, our comparative analysis revealed that patent bile flow is of greater importance than the extent of excision in the long-term treatment efficacy of bile duct cysts, particularly for Type IVa cysts.

Long-term postoperative complications, particularly recurrent cholangitis and pancreatitis, are associated with postoperative cancer [29, 30]. It is widely believed that postoperative cancer of bile duct cysts is attributable to failure of radical resection. According to this notion, postoperative carcinogenesis occurs at the remaining cystic dilatation of the lesioned bile ducts. However, the notion is questionable for the following reasons. First of all, there is no clear histological boundary between the lesioned and normal bile duct; the so-called pathological radical resection is frequently defined by eyes and therefore arbitrary [31]. Second, the pathogenesis of pre- and postoperative carcinogenesis of bile duct cysts is completely different. Preoperative cancer is the result of the abnormal confluent pancreatic juice that erodes the bile duct, while postoperative carcinogenesis is caused by recurrent cholangitis due to obstructed bile flow [32, 33]. Postoperatively, the cause of preoperative cancer no longer exists because the biliary and pancreatic drainage are now separated, so erosion of the bile duct by pancreatic juice no longer exists. In other words, after surgery the mechanism of carcinogenesis has changed. Third, the high incidence of postoperative cancer after incomplete resection should be attributable to the failure to establish patent bile flow. As proper bile flow is easier to achieve in patients who had radical resection, the lower cancer rate may not be the result of complete resection.

In our data, there were only 4 cases of postoperative cancer during the follow-up period. All 4 patients did not have a proper bile flow, even though 3 had radical resection of the cysts. These data strengthen the importance of patent bile flow in the prevention of cancer in these patients. In contrast, patients who had their cysts radically removed could still develop cancer. However, due to the small number of cancer cases, we are not yet able perform a statistical analysis on this issue. We will continue to follow-up these patients, and hope to present significant data in the future.

Our study has limitations. It is a retrospective study, and the study was confined to a single institution. In addition, the follow-up time was not long enough to examine the impact of radical excision and proper bile flow on postoperative cancer occurrence.

## Conclusions

We have provided substantial evidence showing that ensuring patent bile flow greatly improved the long-term postoperative outcomes in adult patients with bile duct cysts, particularly in those with Type Ic and IVa cysts, and when complete excision was unachievable. The establishment of proper bile flow, in some cases, exerted a greater impact on long-term outcomes than did complete excision. Thus, establishing proper bile flow can ensure good long-term outcomes in complicated cases of bile duct cysts and when complete excision is not feasible. The improved surgical treatment approach, if further substantiated, can be expected to widely benefit adult patients with bile duct cysts, decreasing the likelihood of reoperation, and reducing the incidence of late complications and malignancy. Nevertheless, it should be noted that complete excision has been an essential surgical approach for bile duct cyst patients, and should still be achieved whenever possible.

## Additional file

Additional file 1: **Table S1.** Relationship of surgery types and clinical outcomes. **Table S2.** Recent complications. **Table S3.** Grading of recent complications. **Table S4.** oncological follow up. (DOCX 15 kb)

## Abbreviations

ALP: alkaline phosphatase
ALT: alanine aminotransferase
AST: aspartate aminotransferase
BDC: bile duct cyst
CC: choledochal cyst
GGT: gamma-glutamyl transferase
WBC: white blood cell count
