# Supplementary material for: Proper bile duct flow, rather than radical excision, is the most critical factor determining treatment outcomes of bile duct cysts
Source: BMC Gastroenterol. 2018 Aug 23;18:129. doi: 10.1186/s12876-018-0862-3 (PMC6107957; doi:10.1186/s12876-018-0862-3)
Supplement: Supplementary file 1 — Table S1. Relationship of surgery types and clinical outcomes. Table S2. Recent complications. Table S3. Grading of recent complications. Table S4. oncological follow up. (DOCX 15 kb) [file 12876_2018_862_MOESM1_ESM.docx]

**Table S1**: Relationship of surgery types and clinical outcomes

|  | Surgery I | Surgery II |
| --- | --- | --- |
| Late complications | 9 | 11 |
| Long-term biliary function |  |  |
| Excellent | 41 | 56 |
| Good | 1 | 8 |
| Fair | 7 | 6 |
| Poor | 1 | 4 |

**Table S2**: Recent complications

| Type of cyst | Recent complications | | | |  |
| --- | --- | --- | --- | --- | --- |
|  | Bile leakage | Pancreatic leakage | Abdominal hemorrhage | Abdominal infection | Incision delayed healing |
| I | 13 | 3 | 2 | 10 | 7 |
| IVa | 14 | 2 | 2 | 8 | 6 |

**Table S3**: Grading of recent complications

| Type of cyst | grade of complications | | | |  |
| --- | --- | --- | --- | --- | --- |
|  | I | II | IIIa | IIIb | Total |
| I | 10 | 7 | 2 | 1 | 20 |
| IVa | 8 | 6 | 1 | 2 | 17 |

**Table S4**: oncological follow up

| Patient # | Type of cyst | radical resection | proper bile flow | time of cancer after surgery (months) | treatment |
| --- | --- | --- | --- | --- | --- |
| 1 | Ia | Yes | No | 63 | conservative |
| 2 | Ia | Yes | No | 88 | conservative |
| 3 | IVa | Yes | No | 72 | conservative |
| 4 | IVa | No | No | 46 | conservative |
